# Machine learning-based risk prediction for major adverse cardiovascular events in a Brazilian hospital: Development, external validation, and interpretability

**DOI:** 10.1371/journal.pone.0311719

**Published:** 2024-10-11

**Authors:** Gilson Yuuji Shimizu, Michael Schrempf, Elen Almeida Romão, Stefanie Jauk, Diether Kramer, Peter P. Rainer, José Abrão Cardeal da Costa, João Mazzoncini de Azevedo-Marques, Sandro Scarpelini, Katia Mitiko Firmino Suzuki, Hilton Vicente César, Paulo Mazzoncini de Azevedo-Marques

**Affiliations:** 1 Ribeirão Preto Medical School, University of São Paulo, Ribeirão Preto, São Paulo, Brazil; 2 Steiermärkische Krankenanstaltengesellschaft m. b. H., Graz, Austria; 3 Medical University of Graz, Graz, Austria; 4 Predicting Health GmbH, Graz, Austria; 5 Division of Cardiology, Medical University of Graz, Graz, Austria; Faculdade de Medicina de São José do Rio Preto, BRAZIL

## Abstract

**Background:**

Studies of cardiovascular disease risk prediction by machine learning algorithms often do not assess their ability to generalize to other populations and few of them include an analysis of the interpretability of individual predictions. This manuscript addresses the development and validation, both internal and external, of predictive models for the assessment of risks of major adverse cardiovascular events (MACE). Global and local interpretability analyses of predictions were conducted towards improving MACE’s model reliability and tailoring preventive interventions.

**Methods:**

The models were trained and validated on a retrospective cohort with the use of data from Ribeirão Preto Medical School (RPMS), University of São Paulo, Brazil. Data from Beth Israel Deaconess Medical Center (BIDMC), USA, were used for external validation. A balanced sample of 6,000 MACE cases and 6,000 non-MACE cases from RPMS was created for training and internal validation and an additional one of 8,000 MACE cases and 8,000 non-MACE cases from BIDMC was employed for external validation. Eight machine learning algorithms, namely Penalized Logistic Regression, Random Forest, XGBoost, Decision Tree, Support Vector Machine, k-Nearest Neighbors, Naive Bayes, and Multi-Layer Perceptron were trained to predict a 5-year risk of major adverse cardiovascular events and their predictive performance was evaluated regarding accuracy, ROC curve (receiver operating characteristic), and AUC (area under the ROC curve). LIME and Shapley values were applied towards insights about model interpretability.

**Findings:**

Random Forest showed the best predictive performance in both internal validation (AUC = 0.871 (0.859–0.882); Accuracy = 0.794 (0.782–0.808)) and external one (AUC = 0.786 (0.778–0.792); Accuracy = 0.710 (0.704–0.717)). Compared to LIME, Shapley values suggest more consistent explanations on exploratory analysis and importance of features.

**Conclusions:**

Among the machine learning algorithms evaluated, Random Forest showed the best generalization ability, both internally and externally. Shapley values for local interpretability were more informative than LIME ones, which is in line with our exploratory analysis and global interpretation of the final model. Machine learning algorithms with good generalization and accompanied by interpretability analyses are recommended for assessments of individual risks of cardiovascular diseases and development of personalized preventive actions.

## Introduction

Cardiovascular disease (CVD) remains the leading cause of death in Brazil and worldwide, accounting for approximately 18 million annual deaths [1, 2]. As the population ages and risk factors prevention and health care are no longer effective in many locations, the costs associated with CVD increase, highlighting a growing need for prevention, especially in developing countries, where resources are more limited [3–5].

Risk scores, such as Framingham Risk Score [6], are a way to measure CVD risks. Although simple and easy to use, they consider only linear relationships with few variables and no score has been developed specifically for Brazil [6–9].

Machine learning (ML) models [10, 11] have emerged as a promising alternative to traditional risk scores because of their ability to account for complex relationships between variables and handle large amounts of information extracted from electronic health records (EHRs). According to Weng et al [12], they can provide better predictions, improving up to +3.6% AUC (area under the receiver operating characteristic) measurements, and Queseda et al [13] analyzed 15 different ML models, of which 10 outperformed traditional risk scores. Some authors have focused on major adverse cardiovascular events due to the higher mortality risk associated with them [14–16]. A meta-analysis conducted by Bosco et al. [17] revealed the diagnostic codes adopted to define major adverse cardiovascular events vary widely among observational studies; however, acute myocardial infarction and stroke are the most commonly used MACE components. Definitions using more than 3 points are also common.

An important aspect of an ML model is its ability to generalize to new data sets or other populations. However, a common problem is overfitting, i.e., the model performs well only in the training set. Towards avoiding it, the data set is typically divided into two parts, of which the first is used to train the model and the second is used for validation. Some authors suggest other forms of validation in addition to the internal one (e.g., temporal or external validation) [18]. According to Staffa et al. [19], external validation is the most rigorous form of model validation and should be performed whenever possible. Despite its importance, it remains understudied—in a survey of 84,032 studies of prediction models, only 5% reported its adoption [18].

The reliability of ML models for the end user is another barrier for implementation in clinical settings. Unlike linear models, most ML ones are considered black boxes in the sense they are not interpretable. Local interpretability approaches such as LIME (Local Interpretable Model-Agnostic Explanations) and Shapley values [20–22] can explain the predictions of a given instance. Some studies have successfully adopted them to interpret ML models and, thus, increase confidence in CVD predictions [23–25]. Although such methods are important for finding personalized preventive actions for each patient, few studies have focused on the issue in CVD prediction.

This paper addresses the development and validation of ML models for the prediction of MACE risk. Their generalizability was assessed through external validation in a different population and local interpretability methods were explored towards increases in confidence in predictions and creation of personalized preventive actions for each patient.

## Methods

### Data description

The data used were provided by Ribeirão Preto Medical School, University of São Paulo, Brazil, which is the largest public hospital in the region, with EHRs for more than 1.3 million patients and more than 25,000 annual admissions—12,000 admissions from 2009 to 2022 were included in the study cohort. Only patients older than 18 years were involved.

MIMIC IV dataset [26] with EHRs of more than 299,000 patients from Beth Israel Deaconess Medical Center, USA, was used for the external validation of the models trained with RPMS data. A sample with 16,000 admissions was considered for the validation cohort, in a time window of same length as the RPMS cohort.

### Labels

**Fig 1:**
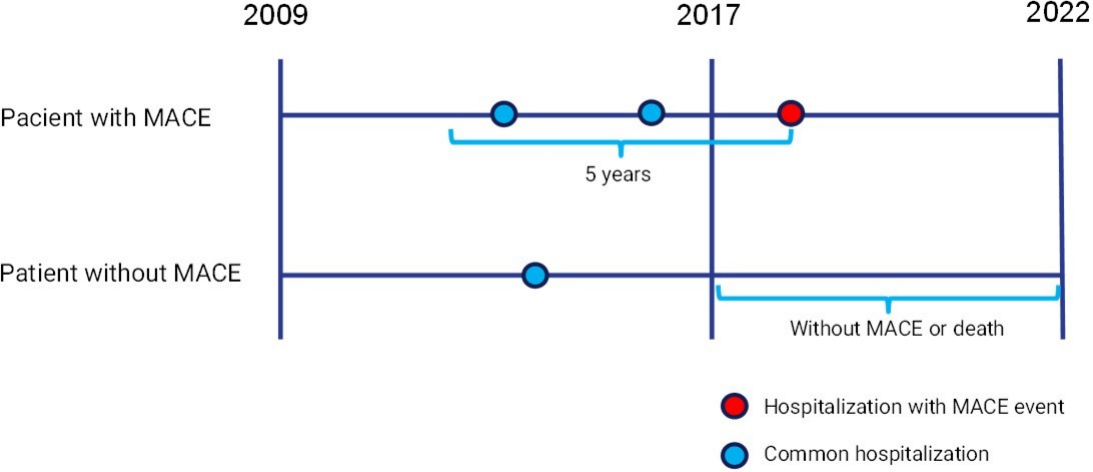
Cohort scheme.

The International Classification of Diseases (ICD-10) was adopted to classify patients with MACE (case group) according to the composition used by Schrempf et al [14]. (see Table 1 for the ICD-10 codes that defined MACE). Only a patient’s first MACE was considered and all previous hospitalizations within a 5-year window were considered MACE. The control group (non-MACE) involved hospitalizations with no MACE and no death within a 5-year window (between 2017 and 2022). See Fig 1 for cohort scheme details. A balanced sample of 6,000 MACE cases and 6,000 non-MACE ones was constructed with data from RPMS for training and internal validation purposes. Another balanced MIMIC IV sample of 8,000 MACE cases and 8,000 non-MACE cases was used for external validation.

**Table 1:** ICD-10 codes for MACE definition.

ICD-10	Description	Type
I20	Angina pectoris	Coronary heart disease
I21	Acute myocardial infarction	
I24	Other acute ischaemic heart diseases	
I46	Cardiac arrest	
I63	Cerebral infarction	Cerebrovascular disease
I64	Stroke, not specified as haemorrhage or infarction	
I71	Aortic aneurysm and dissection	Other cardiovascular disease
I74	Arterial embolism and thrombosis	

### Features

The training cohort and modelling features were defined within the consortium of ERA PerMed and its project partners. Data preprocessing was in alignment with the preprocessing scripts of the Austrian project partner and Schrempf et al [14].

**Fig 2:**
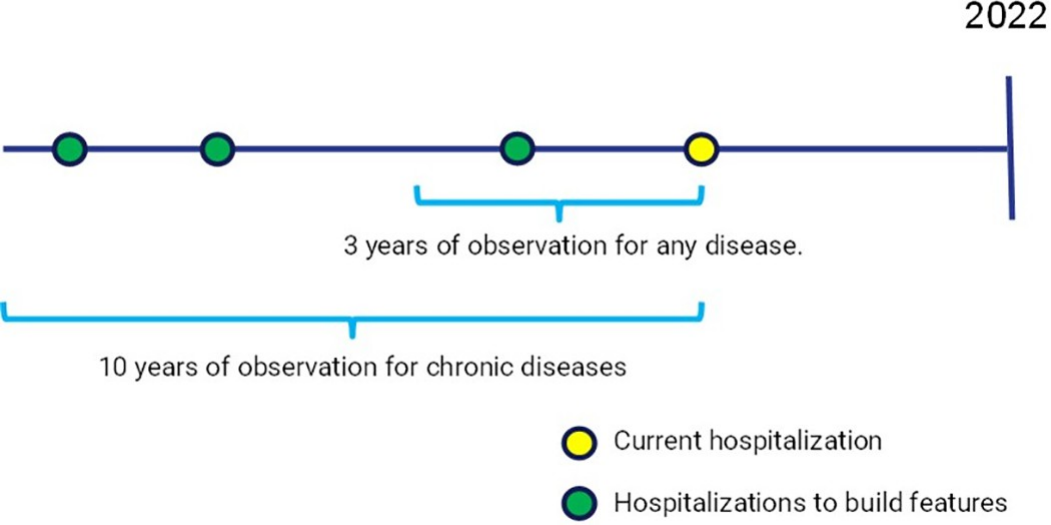
Feature construction scheme.

Demographic and diagnostic attributes were used, totaling 1367 variables, and the experience of Schrempf et al. [14] was the basis for the creation of features based on ICD-10 diagnoses (see Table 2). For each patient in the cohort, a history of up to 10 years of hospitalizations was used for the construction of features based on the diagnoses. Note for features that consider number of diagnoses (e.g., number of diagnoses), the same diagnosis may be counted more than once in the course of past hospitalizations. Features that consider the time since the last diagnosis of a disease use the admission date of the current hospitalization as the reference. See more details about the feature construction in Fig 2.

**Table 2:** Variables tested in the ML algorithms.

Features	Type
Gender	Demographic
Age	
Weeks since last ICD-10 diagnosis (in 3 years)	Diagnoses
Charlson weighted comorbidity score [27]	
Number of diagnoses in each ICD-10 chapter (in 3 years)	
Number of diagnoses in each ICD-10 group (in 3 years)	
Number of diagnoses of chronic diseases (in 10 years)	
Number of diagnoses (in 3 years)	

Charlson weighted comorbidity score [27] measures a patient’s risk of death considering a set of comorbidities. A null score indicates no comorbidity has been found, whereas a high one denotes a high risk of mortality. R comorbidity package [28] was used to calculate the score.

Due to the large number of variables, Boruta method [29] was adopted for excluding irrelevant attributes prior to the training of the models. It uses the importance generated by Random Forest algorithm [30] and performs a statistical test to select relevant variables. At the end of the process, 55 attributes were selected.

### Machine learning algorithms

Eight classes of ML algorithms, namely Penalized Logistic Regression, Random Forest, XGBoost, Decision Tree, Support Vector Machine, k-Nearest Neighbors, Naive Bayes, and Multi-Layer Perceptron were used [10, 11] due to their popularity and good performance in classification problems.

Towards a comparison of the methods´ performances, 70% of the samples were randomly selected for training the models and 30% were used for validation. The procedure, called data splitting, is important, for it simulates the application of a model to a new independent data set. The hyperparameters of the models were determined through 10-fold cross-validation, with a grid of values for the identification of the best ones. The models were developed in the training sample and applied to a test sample in RStudio software [31] with the use of Tidymodels package (https://cran.r-project.org/package=tidymodels).

### Statistical analysis

Exploratory analyses, such as correlation ones and scatter plots, were performed towards the understanding of the relationship between features and label (MACE) before the models had been trained so that their viability could be assessed and the most relevant features could be detected.

Accuracy measure, F2 score, ROC curve (receiver operating characteristic), AUC measure (area under the ROC curve), and calibration plots were used for evaluations and comparison of the models´ performances in the validation sample—DeLong method obtained confidence intervals [32]. Details of the measures can be found in James et al. [11]. A statistical analysis of the exploratory analysis and evaluation of model performance were conducted with RStudio software.

### Interpretability

Local interpretability methods LIME and Shapley values interpreted individual predictions of the best performing model. LIME fits weighted local linear regressions with penalization, taking into account a noisy sample around the instance to be explained. Therefore, the prediction of an instance is interpreted by the locally estimated coefficients or effects (coefficient × attribute value). Shapley value calculates the contribution of each attribute to the prediction of an individual, considering all possible combinations of attributes. Individuals with high and low predictions were selected and analyzed and interpretations of predictions from a sub-sample were combined to providing a global interpretation of the model.

### Ethical approval

The Institutional Review Board of RPMS’ hospital (Hospital das Clínicas of the Faculty of Medicine of Ribeirão Preto of USP) approved the protocol for this study under Certificate of Presentation of Ethical Appreciation 69493423.6.0000.5440. Only patients older than 18 were involved, and the requirement for informed consent was waived. Data were accessed for research purposes on May 29, 2023. The corresponding author had access to information that could identify individual participants during data collection. Subsequently, all data was anonymized for processing purposes. The MIMIC-IV dataset was provided after completion of the “CITI Data or Specimens Only Research” training and acceptance of the terms of use.

## Results

### Exploratory analysis

**Fig 3:**
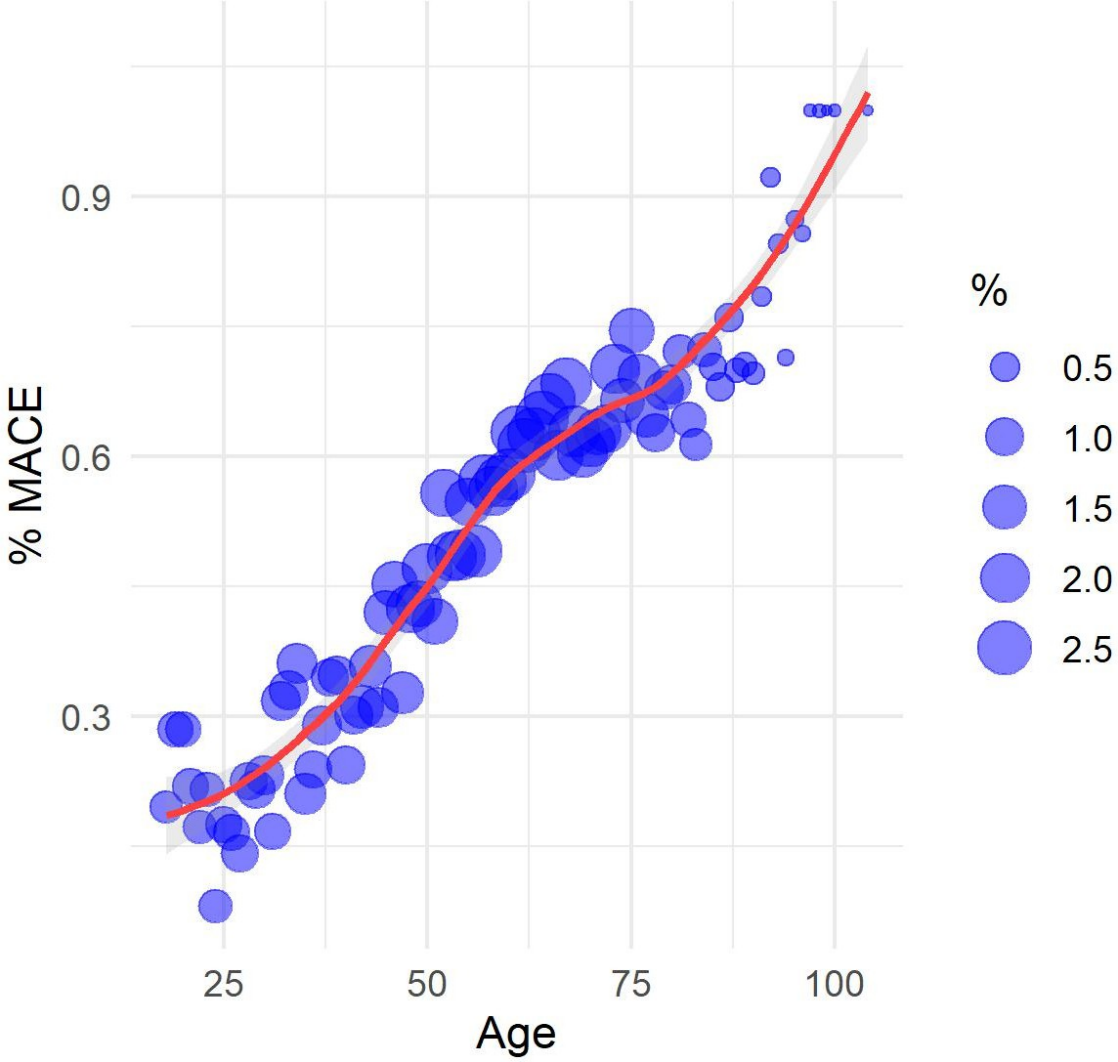
Scatter plot among age and MACE (%).

**Fig 4:**
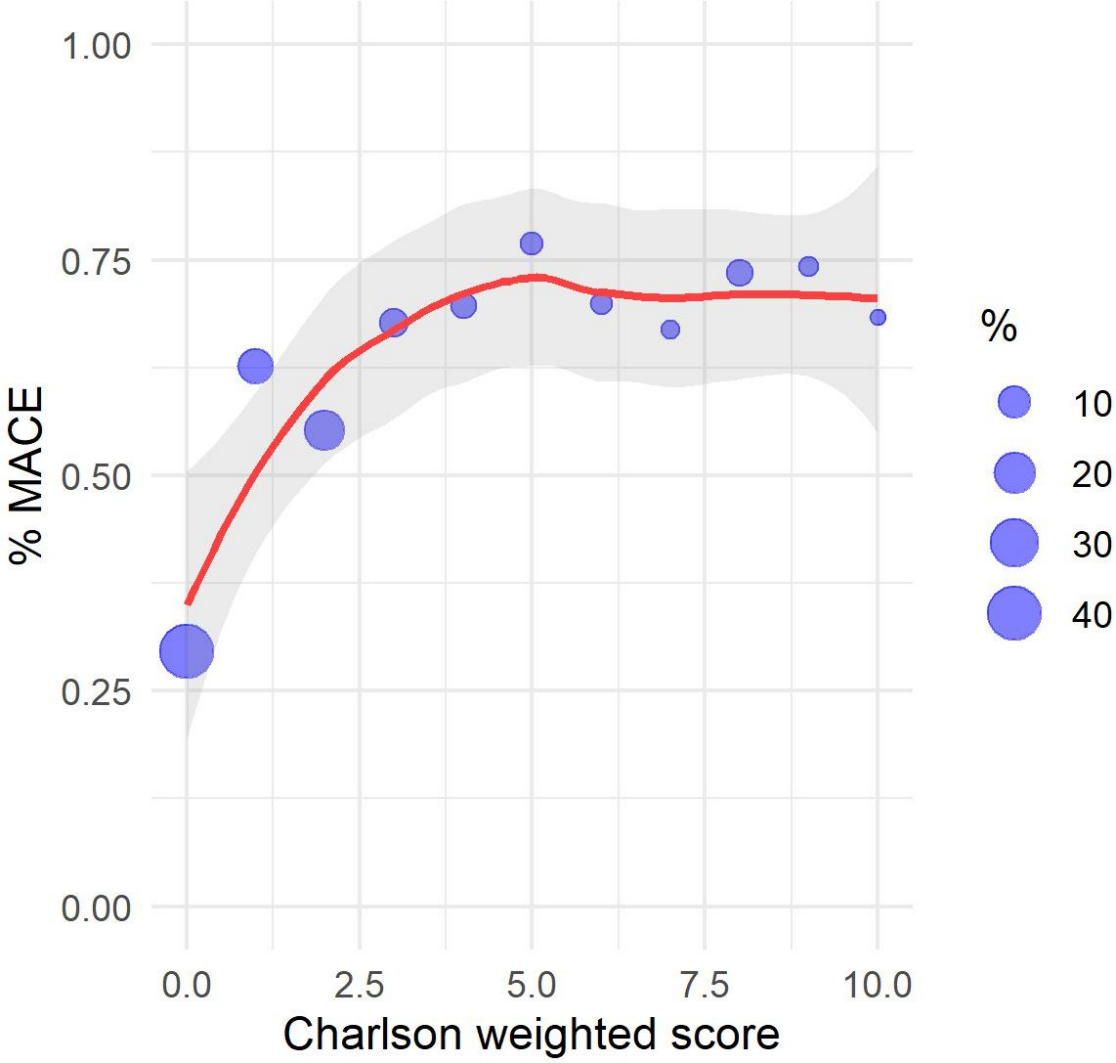
Scatter plot among Charlson score and MACE (%).

**Fig 5:**
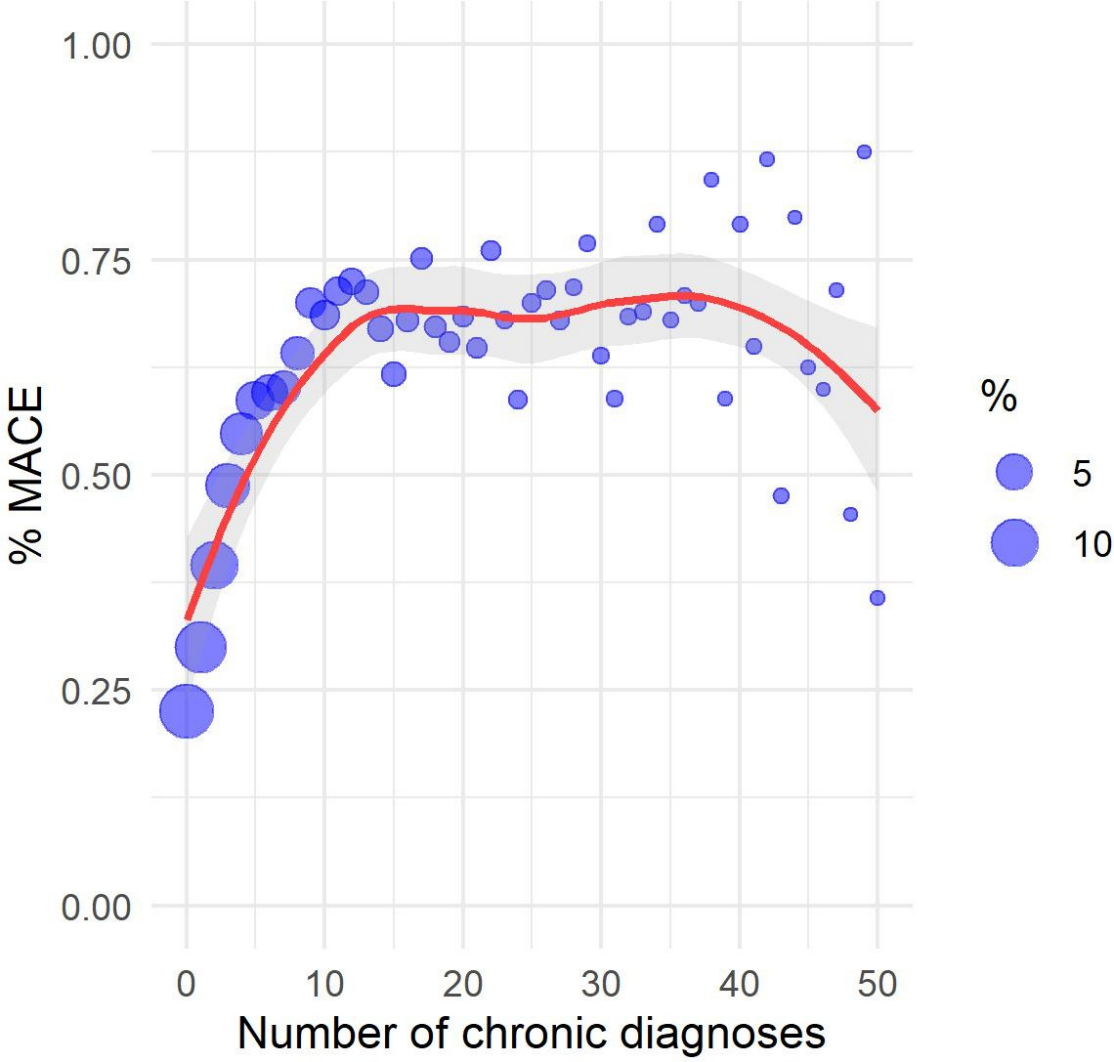
Scatter plot among chronic diagnoses and MACE (%).

**Fig 6:**
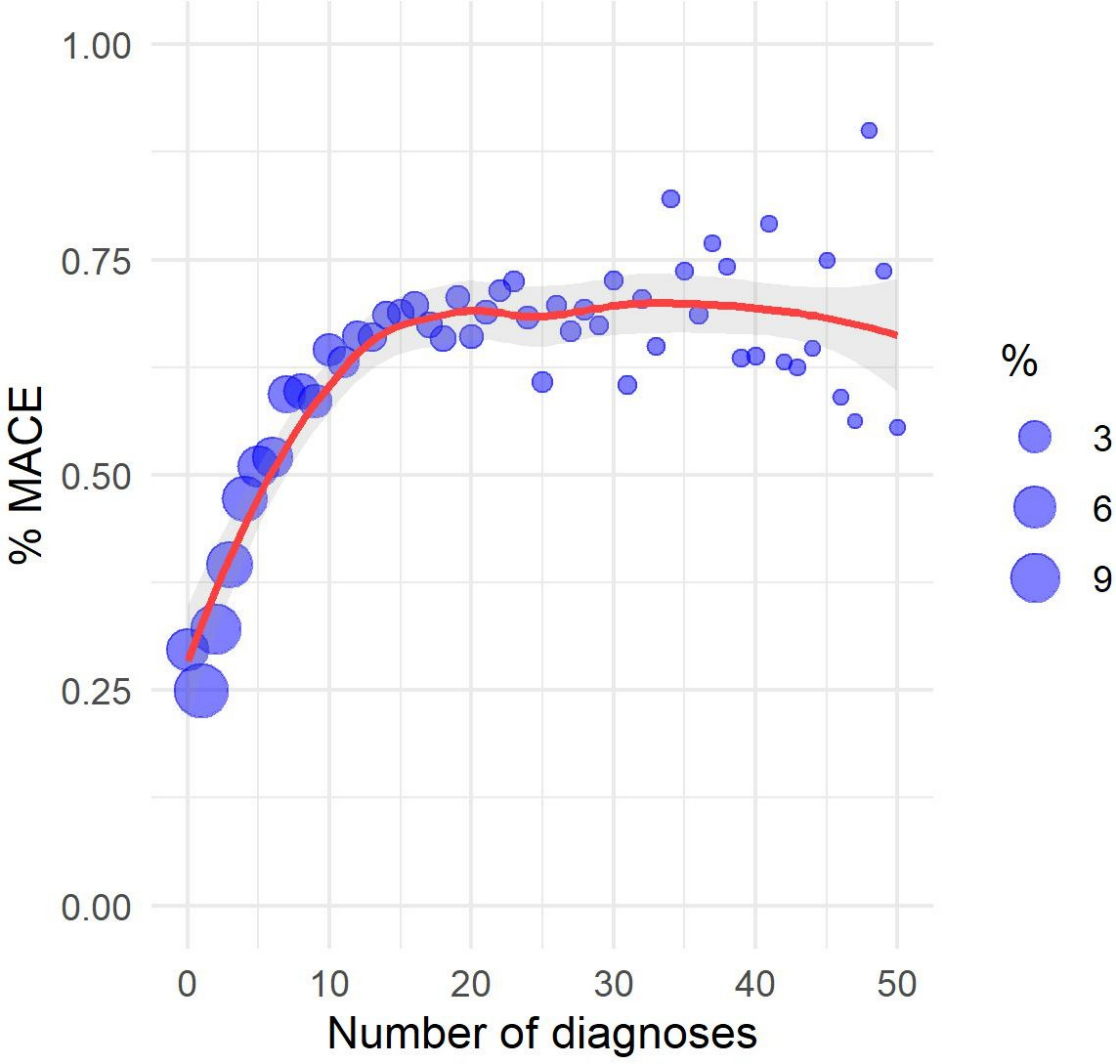
Scatter plot among diagnoses and MACE (%).

The study sample consisted of patients of 53.33 years mean age (17.52 standard deviation). Table 3 compares some descriptive statistics between RPMS and MIMIC IV samples. Some scatter plots were constructed towards the understanding of the relationship between attributes and label (MACE). Figs 3–6 show the scatter plots of age, weighted Charlson score, number of chronic disease diagnoses, and number of diagnoses versus the proportion of patients with a MACE occurrence. Despite a strong linear relationship between age and % of MACE, other characteristics such as weighted Charlson score show a non-linear one with % of MACE.

**Table 3:** Characteristics of patients in the RPMS and MIMIC IV cohorts. P-values were calculated using univariate logistic regression for each variable.

Statistics	RPMS			MIMIC IV		
	MACE (n = 6000)	NON-MACE (n = 6000)	P-value	MACE (n = 8000)	NON-MACE (n = 8000)	P-value
Age average (SD)	60.88 (15.22)	49.80 (17.33)	<0.001	69.96 (14.13)	54.54 (19.00)	<0.001
Gender male	56.13%	48.13%	<0.001	57.53%	46.04%	<0.001
Charlson score average (SD)	2.78 (2.88)	1.29 (2.13)	<0.001	3.54 (2.97)	2.00 (2.73)	<0.001
ICD-10 chap. 01: Certain infectious and parasitic diseases	24.82%	8.68%	<0.001	20.11%	16.65%	<0.001
ICD-10 chap. 02: Neoplasms	36.58%	29.08%	<0.001	15.07%	16.44%	0.017
ICD-10 chap. 03: Diseases of the blood and blood-forming organs and disorders involving the immune mechanism	12.42%	4.62%	<0.001	39.67%	25.55%	<0.001
ICD-10 chap. 04: Endocrine, nutritional and metabolic diseases	35.52%	15.87%	<0.001	81.70%	53.44%	<0.001
ICD-10 chap. 05: Mental and behavioural disorders	18.00%	7.07%	<0.001	37.66%	43.42%	<0.001
ICD-10 chap. 06: Diseases of the nervous system	8.52%	5.92%	<0.001	36.16%	29.89%	<0.001
ICD-10 chap. 07: Diseases of the eye and adnexa	3.10%	3.88%	0.020	9.46%	5.03%	<0.001
ICD-10 chap. 08: Diseases of the ear and mastoid process	0.92%	2.05%	<0.001	2.14%	1.64%	0.021
ICD-10 chap. 09: Diseases of the circulatory system	74.93%	22.43%	<0.001	94.44%	53.30%	<0.001
ICD-10 chap. 10: Diseases of the respiratory system	24.55%	9.87%	<0.001	37.32%	24.59%	<0.001
ICD-10 chap. 11: Diseases of the digestive system	28.85%	18.18%	<0.001	44.92%	38.83%	<0.001
ICD-10 chap. 12: Diseases of the skin and subcutaneous tissue	6.47%	5.15%	0.002	12.52%	9.87%	<0.001
ICD-10 chap. 13: Diseases of the musculoskeletal system and connective tissue	9.15%	11.80%	<0.001	35.31%	26.80%	<0.001
ICD-10 chap. 14: Diseases of the genitourinary system	27.37%	18.37%	<0.001	51.21%	27.33%	<0.001
ICD-10 chap. 15: Pregnancy, childbirth and the puerperium	0.13%	1.88%	<0.001	0.15%	6.61%	<0.001
ICD-10 chap. 16: Certain conditions originating in the perinatal period	0.02%	0.07%	0.215	0.00%	0.01%	0.930
ICD-10 chap. 17: Congenital malformations, deformations and chromosomal abnormalities	1.27%	1.58%	0.144	4.04%	2.53%	<0.001
ICD-10 chap. 18: Symptoms, signs and abnormal clinical and laboratory findings, not elsewhere classified	22.32%	8.28%	<0.001	50.83%	47.06%	<0.001
ICD-10 chap. 19: Injury, poisoning and certain other consequences of external causes	19.78%	20.77%	0.180	22.29%	19.98%	<0.001
ICD-10 chap. 20: External causes of morbidity and mortality	5.70%	11.48%	<0.001	26.56%	22.94%	<0.001
ICD-10 chap. 21: Factors influencing health status and contact with health services	27.02%	14.20%	<0.001	79.23%	63.39%	<0.001
ICD-10 chap. 22: Codes for special purposes	1.10%	0.08%	<0.001	NA	NA	NA

**Fig 7:**
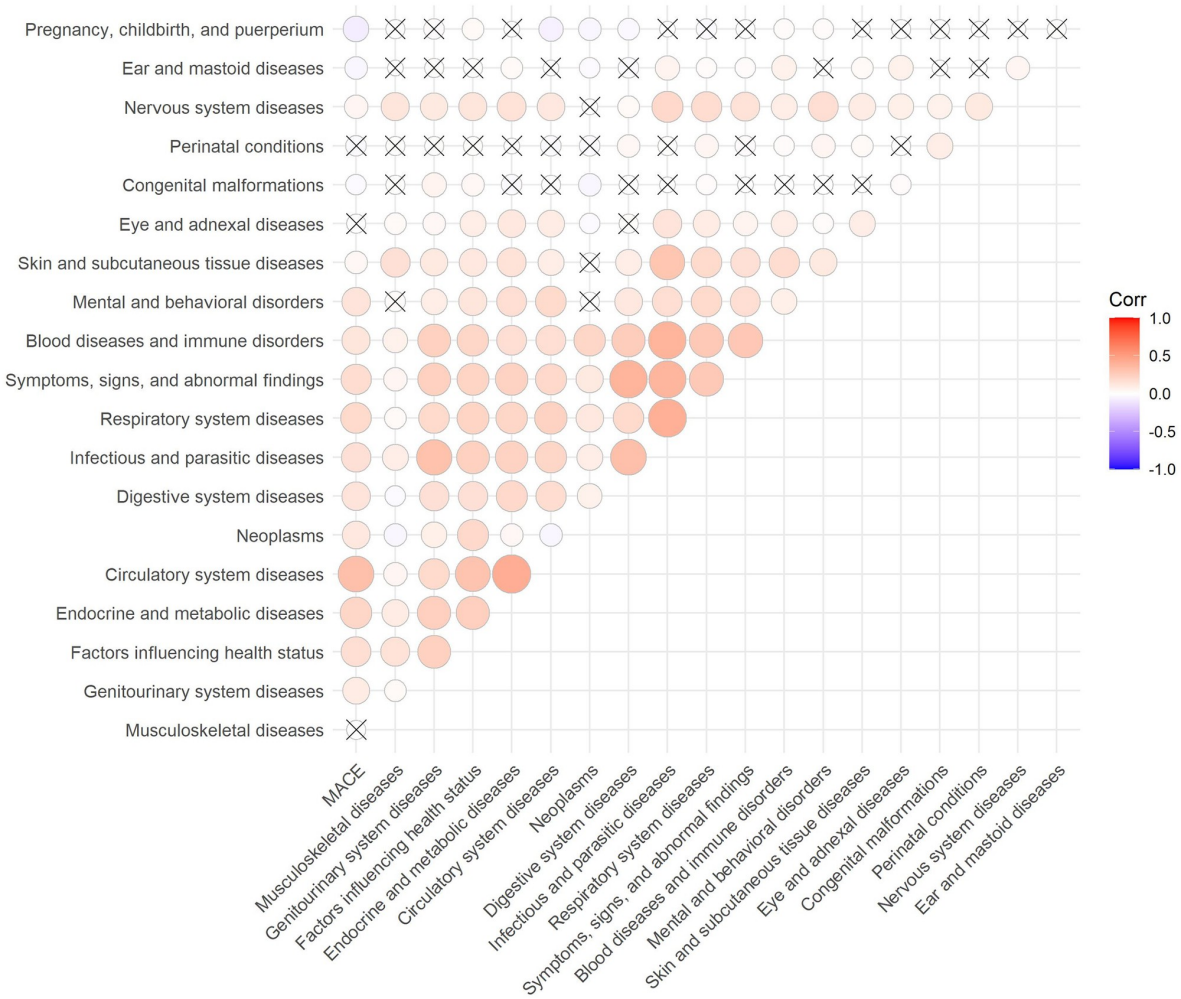
Correlation matrix between ICD-10 chapters and MACE. × represents non-significant correlations (p-value > 5%).

Due to the large number of ICD-10 codes, chapters on ICD-10 codes, rather than each code individually, were analyzed. Fig 7 displays a graph of correlations between ICD-10 chapters and proportion of MACE. As an example, a positive correlation is established between MACE and endocrine and metabolic diseases, and a negative one is observed between MACE and conditions related to pregnancy, childbirth, and puerperium. Note some pairs of chapters have a high correlation, which can hamper the training of traditional linear models due to multicollinearity.

### Internal validation

**Fig 8:**
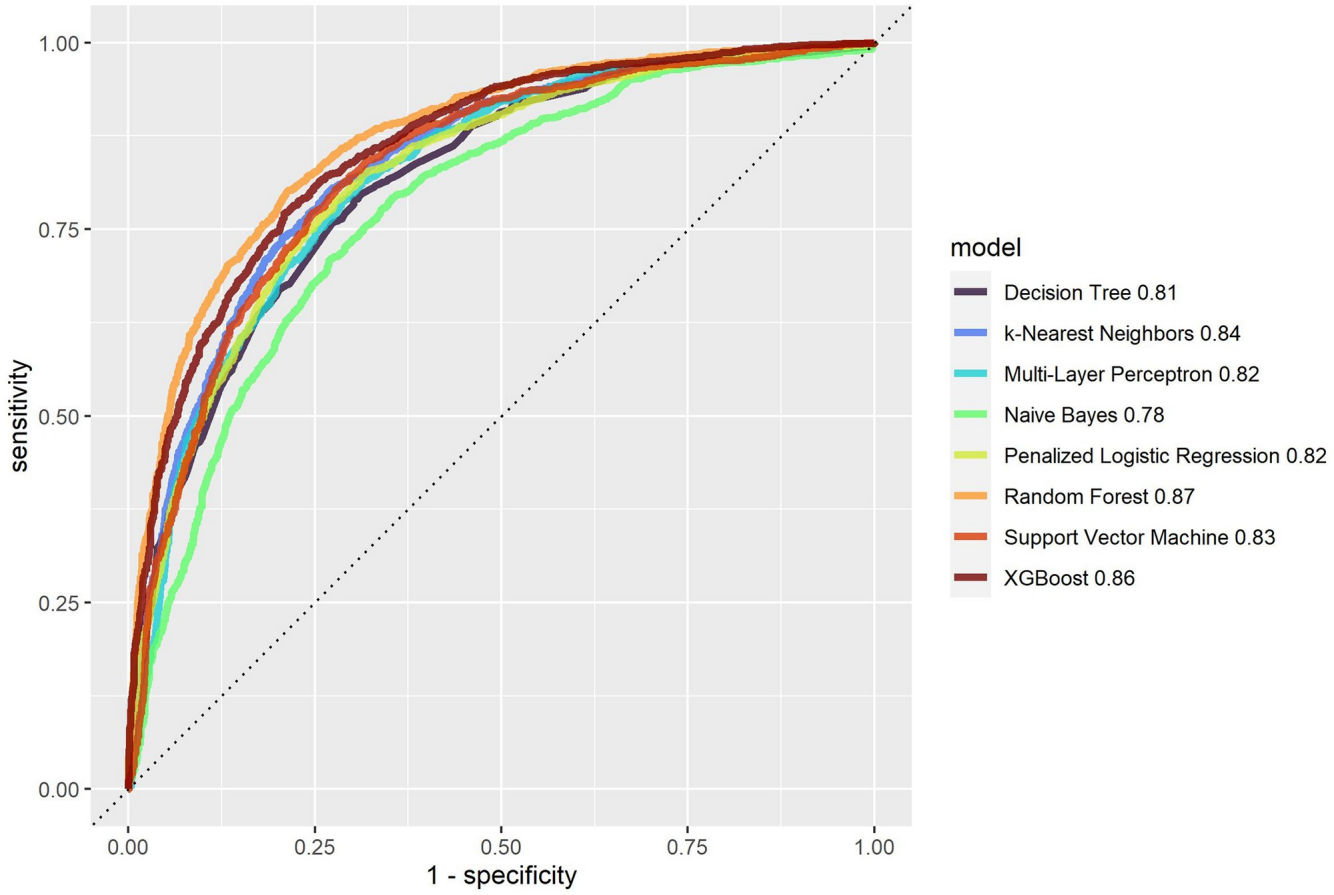
Roc curves for the ML algorithms applied to the test sample.

**Fig 9:**
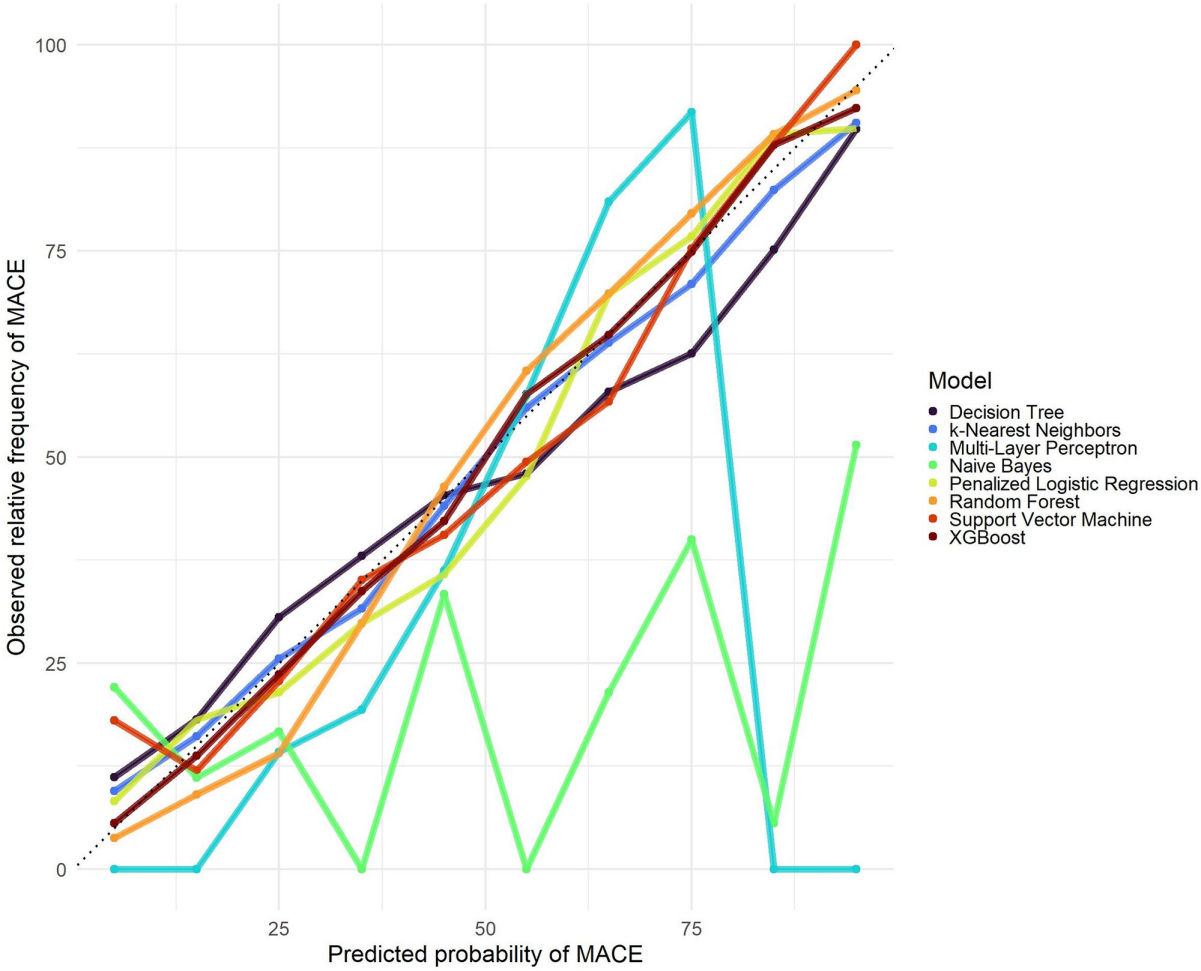
Models’ calibration plots in RPMS sample.

All trained models were applied to the test sample for internal validation. Table 4 shows the AUC and accuracy measures with their respective 95% confidence intervals for all ML algorithms in the test sample and Fig 8 displays a comparison of the ROC curves of all methods. Although all algorithms achieved AUCs higher than 0.779, accuracy higher than 0.720 and F2 score higher than 0.713, Random Forest outperformed them. Except Naive Bayes and Support Vector Machine, all models have a good calibration of predicted and observed values (see the calibration plots in Fig 9).

**Table 4:** Performance of ML algorithms in the test sample according to AUC, accuracy and F2 metrics, with respective 95% confidence intervals.

ML algorithm	AUC (CI 95%)	Accuracy (CI 95%)	F2 score (CI 95%)
Penalized Logistic Regression	0.823 (0.809–0.836)	0.756 (0.746–0.771)	0.778 (0.764–0.791)
Random Forest	0.871 (0.859–0.882)	0.794 (0.782–0.808)	0.818 (0.806–0.831)
XGBoost	0.858 (0.845–0.869)	0.781 (0.768–0.795)	0.793 (0.780–0.806)
Decision Tree	0.813 (0.799–0.827)	0.744 (0.730–0.758)	0.776 (0.762–0.789)
Support Vector Machine	0.813 (0.817–0.844)	0.763 (0.752–0.779)	0.791 (0.777–0.804)
k-Nearest Neighbors	0.837 (0.824–0.850)	0.766 (0.755–0.782)	0.792 (0.778–0.805)
Naive Bayes	0.779 (0.764–0.794)	0.720 (0.709–0.736)	0.713 (0.698–0.728)
Multi-Layer Perceptron	0.823 (0.813–0.840)	0.757 (0.745–0.771)	0.770 (0.756–0.784)

### External validation

**Fig 10:**
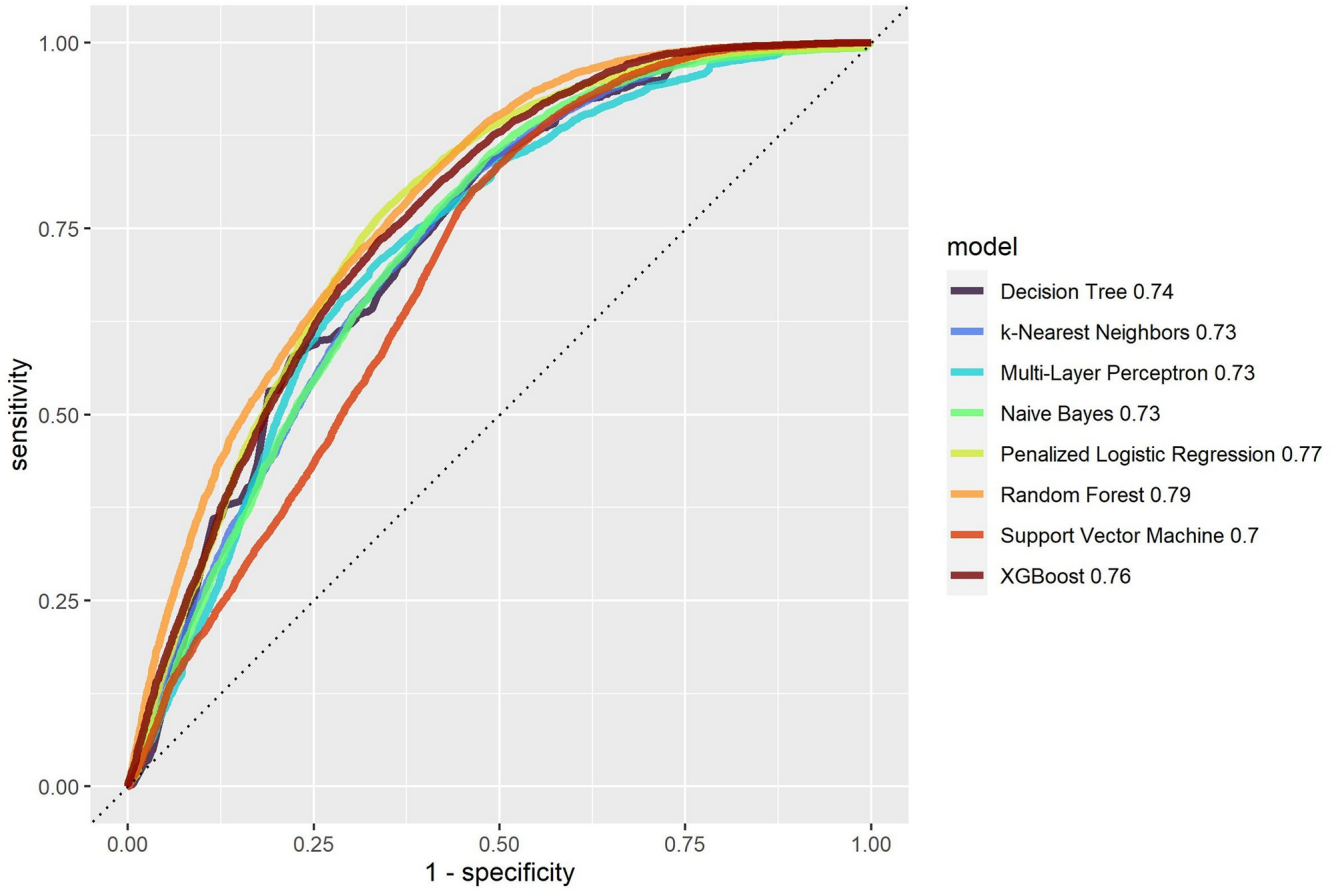
Roc curves for the ML algorithms applied to the MIMIC IV dataset.

**Fig 11:**
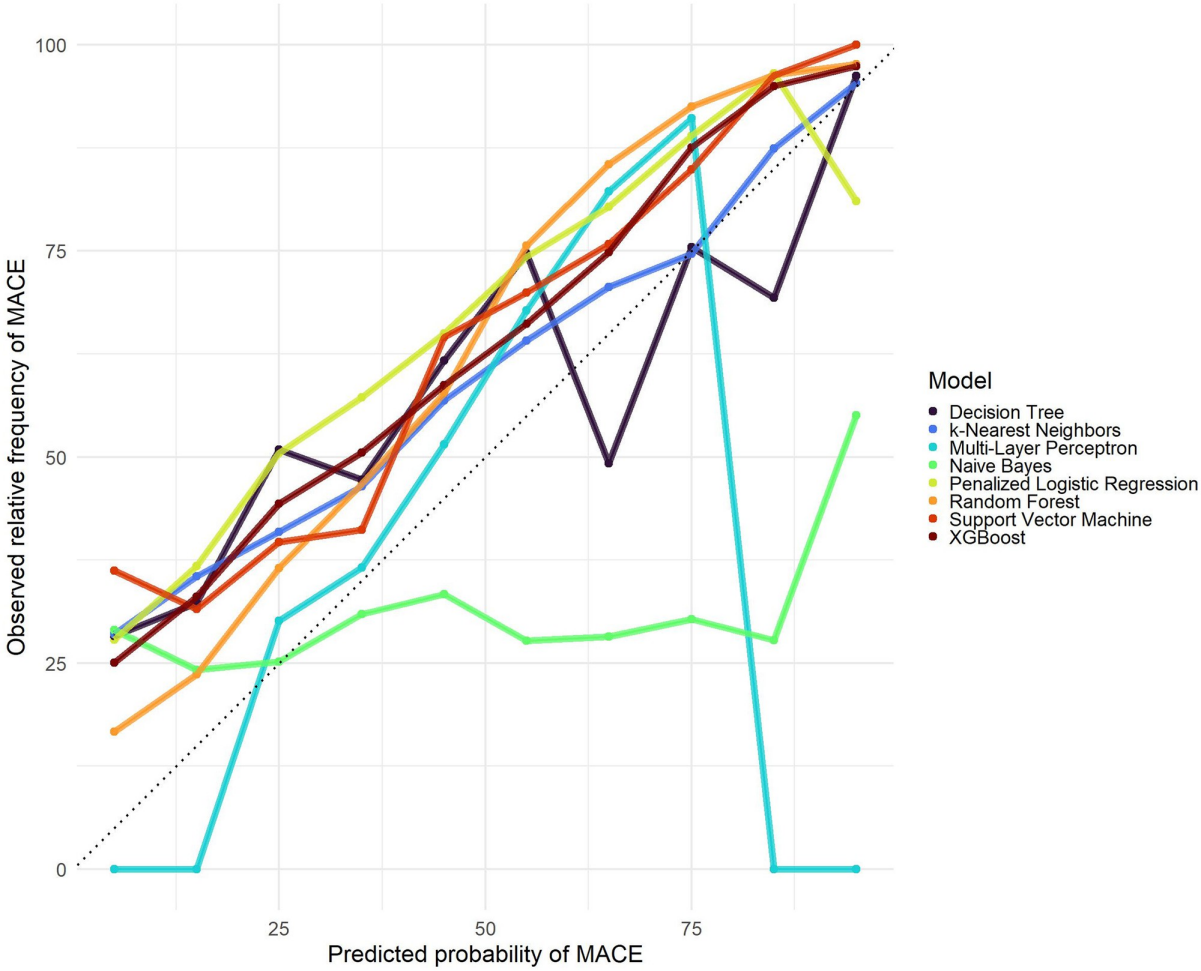
Models’ calibration plots in MIMIC IV sample.

The models developed in the RPMS training sample were applied to a sample of MIMIC IV dataset. The measures of AUC, accuracy, F2 score and ROC curves were again used for comparisons of ML algorithms (Table 5 and Fig 10). All models provided an AUC higher than 0.699, accuracy higher than 0.669 and F2 score higher than 0.761. The model calibration plot (Fig 11) indicates the predictions of the models trained on the RPMC sample underestimate the observed probabilities of MACE in the MIMIC IV sample, which can be explained by the differences found in the descriptive analysis between RPMS and MIMIC IV samples (Table 3). Naive Bayes, Decision Tree, and Multi-Layer Perceptron did not show a good calibration between predicted and observed values. Considering all metrics, Random Forest performed best in the external population.

**Table 5:** Performance of ML algorithms in MIMIC IV dataset according to AUC, accuracy and F2 metrics, with respective 95% confidence intervals.

ML algorithm	AUC (CI 95%)	Accuracy (CI 95%)	F2 score (CI 95%)
Penalized Logistic Regression	0.768 (0.760–0.775)	0.715 (0.709–0.723)	0.757 (0.750–0.763)
Random Forest	0.786 (0.778–0.792)	0.710 (0.704–0.717)	0.783 (0.777–0.789)
XGBoost	0.763 (0.756–0.771)	0.697 (0.691–0.705)	0.768 (0.761–0.774)
Decision Tree	0.738 (0.730–0.745)	0.678 (0.673–0.686)	0.787 (0.781–0.793)
Support Vector Machine	0.699 (0.691–0.708)	0.669 (0.663–0.677)	0.761 (0.754–0.767)
k-Nearest Neighbors	0.733 (0.725–0.740)	0.679 (0.673–0.686)	0.775 (0.769–0.782)
Naive Bayes	0.731 (0.723–0.738)	0.682 (0.676–0.689)	0.796 (0.790–0.802)
Multi-Layer Perceptron	0.729 (0.723–0.738)	0.686 (0.680–0.694)	0.776 (0.770–0.783)

### Interpretability

**Fig 12:**
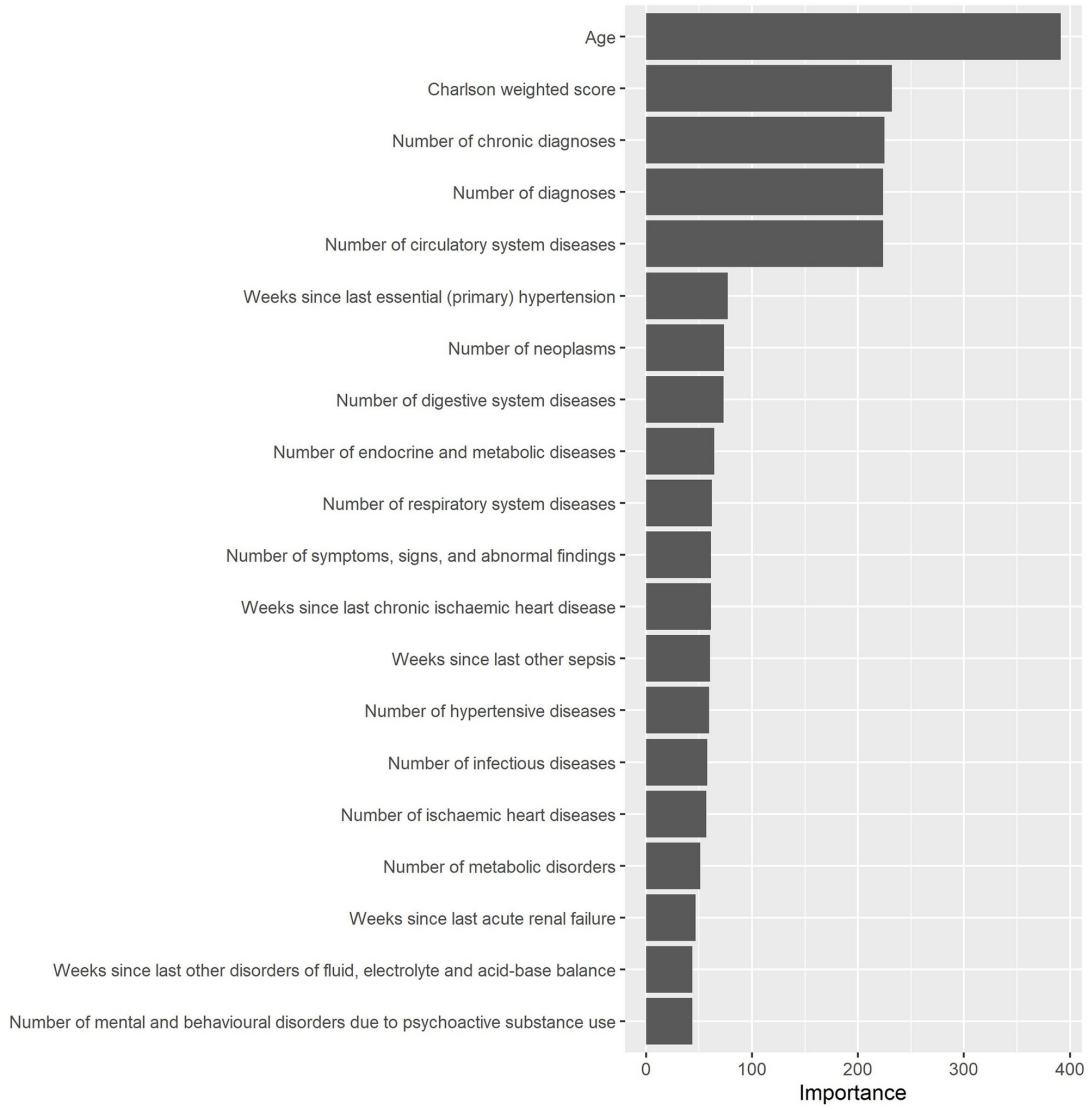
Feature importance of Random Forest.

Since Random Forest performed best, the interpretability analysis focused on its predictions. One of the advantages of Random Forest is it automatically calculates the total contribution of each attribute in the predictions, known as importance. Fig 12 displays the top 20 attributes in Random Forest—age, weighted Charlson score, number of chronic disease diagnoses, number of diagnoses, and number of circulatory system diseases are the most important ones.

**Fig 13:**
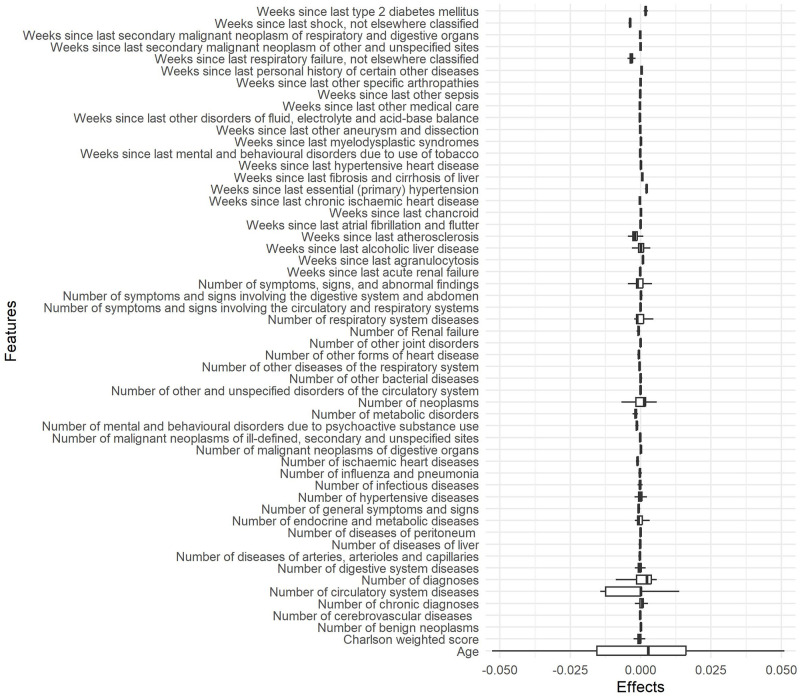
Boxplots of LIME effects for attributes preselected by Boruta method in a subsample of 600 instances.

**Fig 14:**
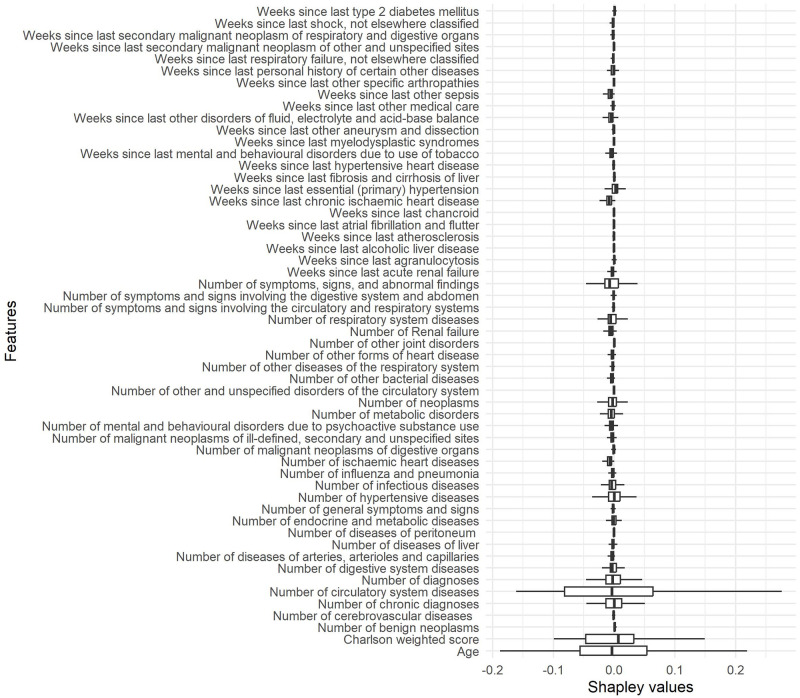
Boxplots of Shapley values for attributes preselected by Boruta method in a subsample of 600 instances.

Another way to interpret a model globally is by considering the local interpretations of a subsample of instances. Figs 13 and 14 show the box plots of LIME and Shapley values effects, respectively, for the attributes preselected by Boruta method, in a subsample of 600 instances. Shapley values captured a larger number of relevant attributes, which is consistent with the importance of Random Forest and exploratory analysis.

**Fig 15:**
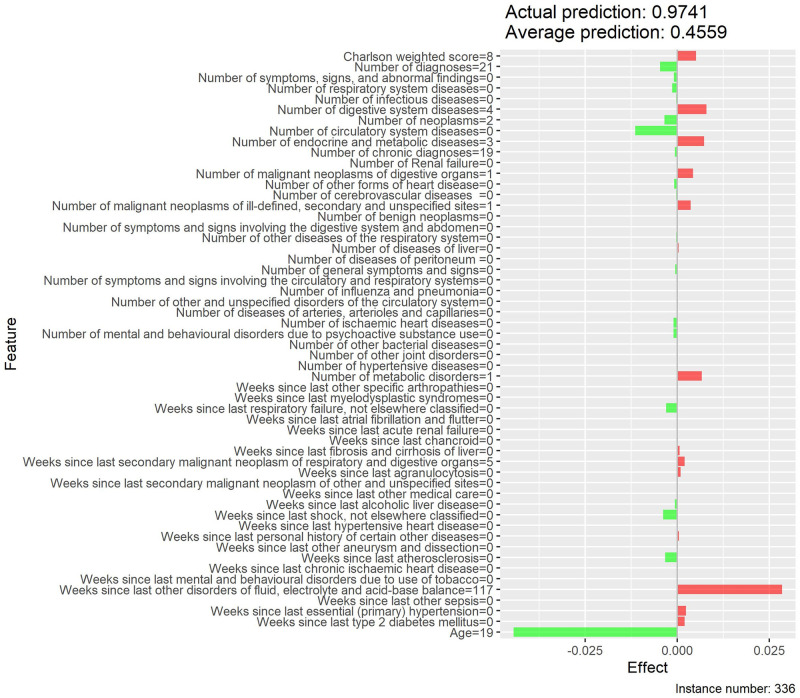
Local interpretability of case with high probability of MACE by LIME effects.

**Fig 16:**
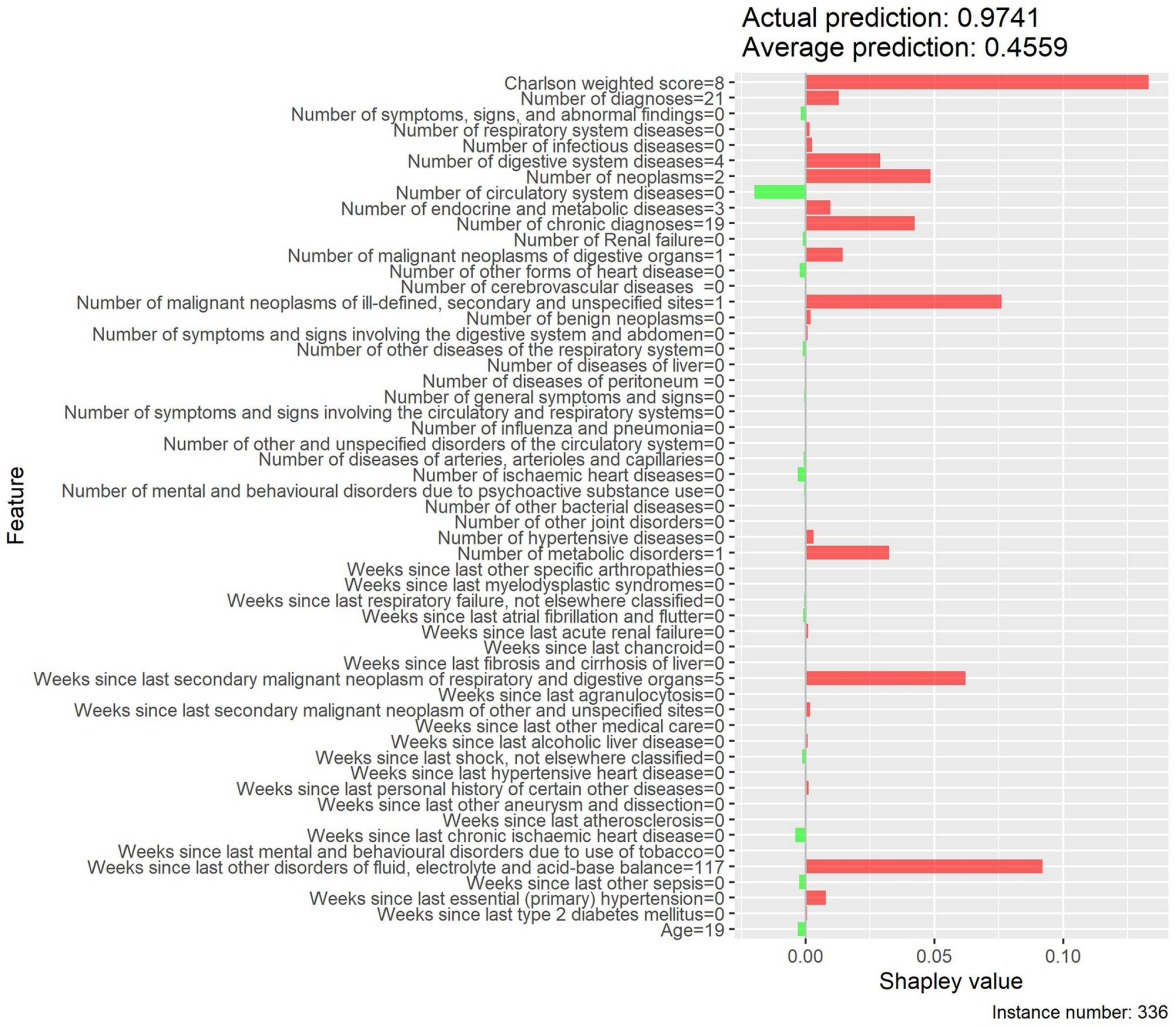
Local interpretability of case with high probability of MACE by Shapley values.

**Fig 17:**
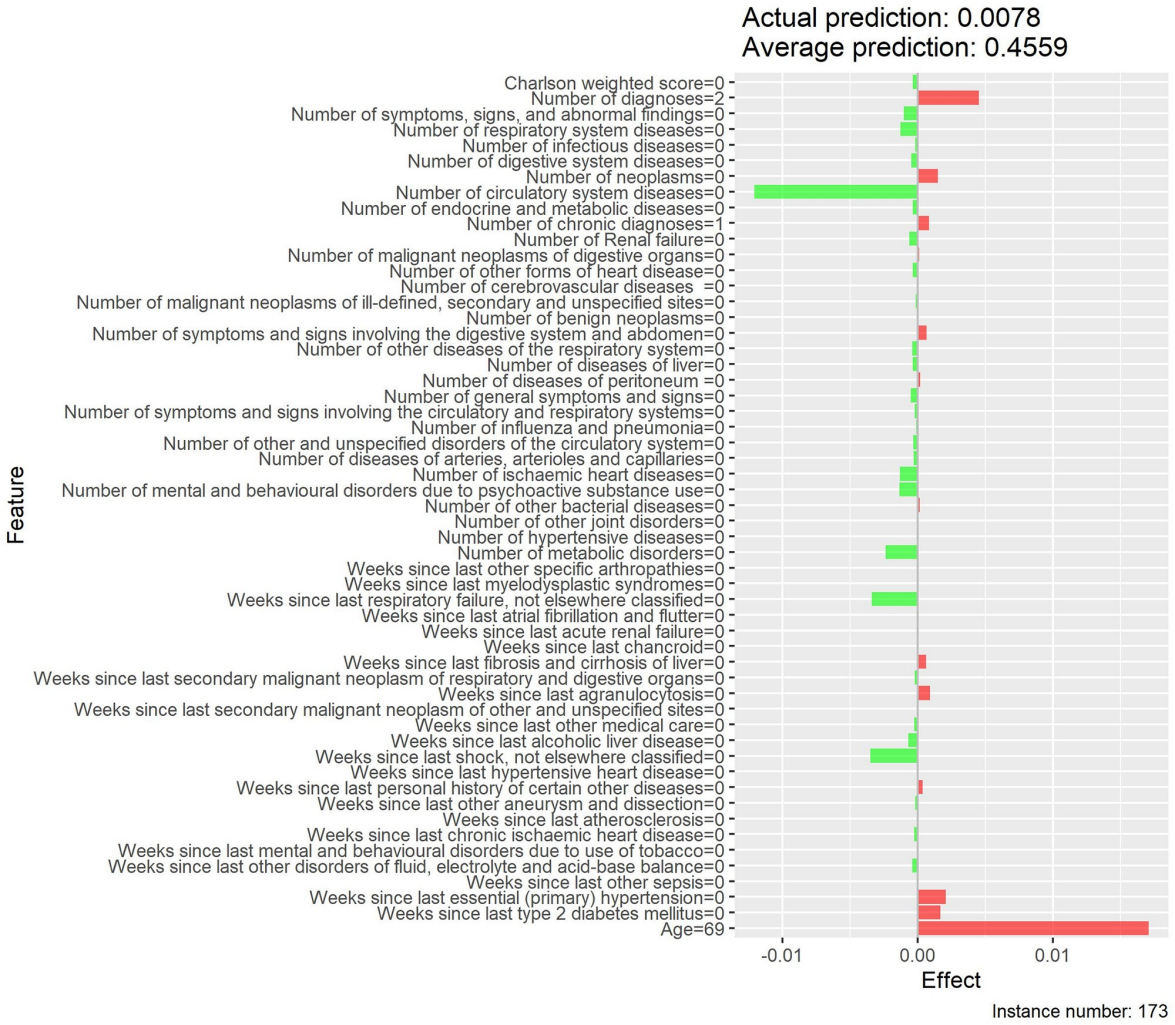
Local interpretability of case with low probability of MACE by LIME effects.

**Fig 18:**
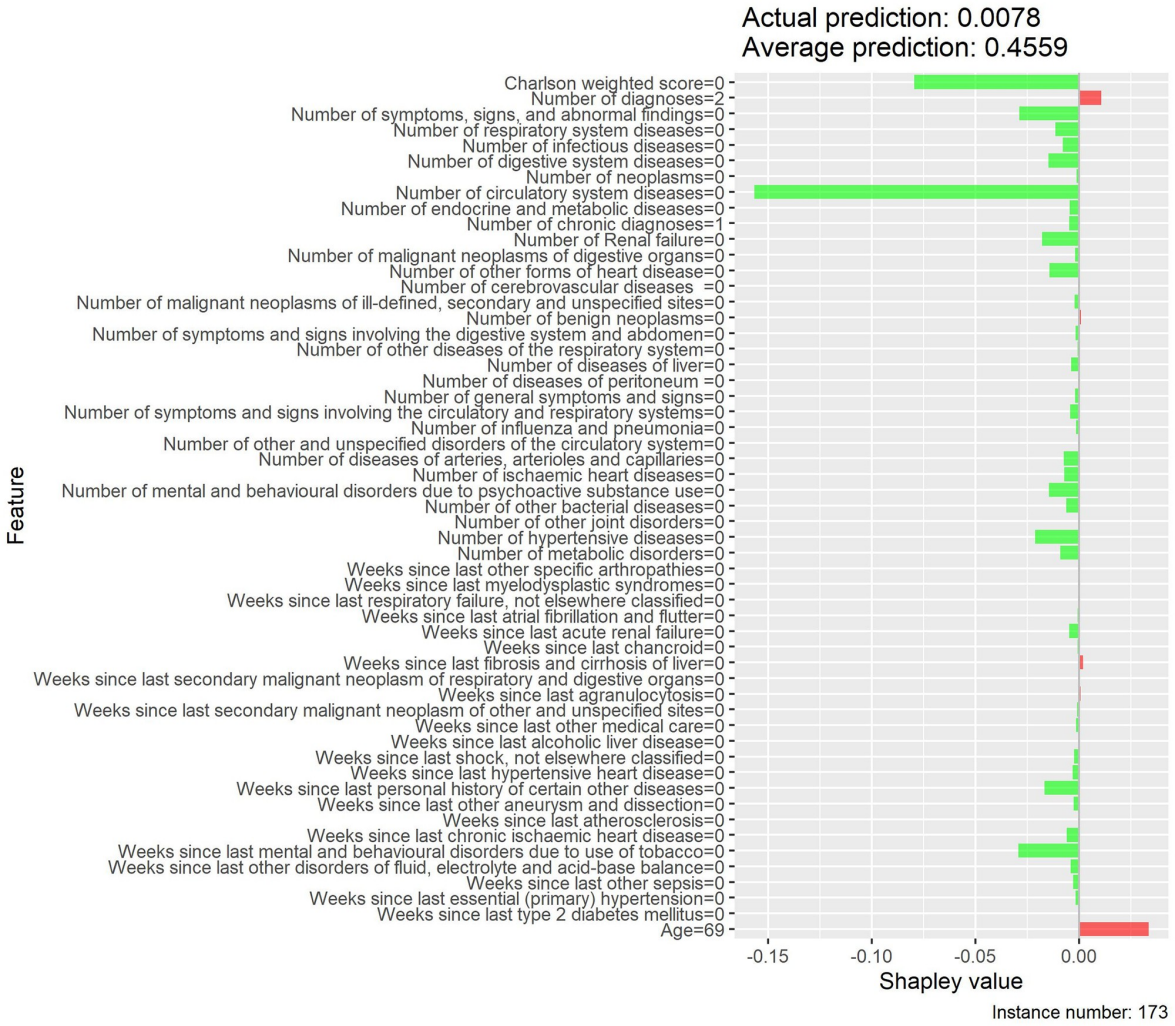
Local interpretability of case with low probability of MACE by Shapley values.

Local interpretability enables the understanding of why a patient is at low or high risk of developing MACE and the contrast of cases with extreme probabilities. Figs 15–18 show a comparison of two extreme cases by LIME and Shapley values—the latter provided explanations consistent with previous results (exploratory analysis and feature importance).

## Discussion

According to the results, among the 8 algorithms tested, Random Forest performed best in identifying the risk of MACE in both RPMS internal validation sample and MIMIC IV external validation one. The results also suggest Shapley values produced better and more detailed explanations of individual predictions than LIME.

The exploratory analysis revealed non-linear relationships between some attributes and percentage of MACE, thus reinforcing algorithms that capture non-linear relationships, such as Random Forest, may be more appropriate to the present problem. ICD-10 chapters with positive correlations with MACE occurrence are diseases of the circulatory system (excluding MACE), endocrine and metabolic diseases, diseases of the respiratory system, abnormal symptoms, factors influencing health status, infectious diseases, diseases of the digestive system, mental disorders, blood diseases, neoplasms, and diseases of the genitourinary system. Note the chapter on abnormal symptoms includes symptoms related to the circulatory system and the chapter on factors influencing health status includes lifestyle problems such as tobacco use, alcohol use, physical inactivity, and poor diet. Descriptive analyses of age, Charlson score, and ICD-10 chapters suggest a higher-risk patient profile in MIMIC IV in comparison to RPMS.

Algorithms based on combinations of decision trees performed best in the internal validation. Random Forest provided 0.871 (0.859–0.882) AUC, 0.794 (0.782–0.808) accuracy and 0.818 (0.806–0.831) F2 score, whereas XGBoost yielded 0.858 (0.845–0.869) AUC, 0.781 (0.768–0.795) accuracy and 0.793 (0.780–0.806) F2 score. Naive Bayes achieved the worst performance, with 0.779 (0.764–0.794) AUC, 0.720 (0.709–0.736) accuracy and 0.713 (0.698–0.728) F2 score.

In the external validation, Random Forest performed best, with 0.786 (0.778–0.792) AUC and 0.710 (0.704–0.717) accuracy, whereas Support Vector Machine achieved the worst performance, with 0.699 (0.691–0.708) AUC and 0.669 (0.663–0.677) accuracy. Such results confirmed the good generalization ability of Random Forest [14, 33, 34]. Although Naive Bayes and Decision Tree have the best F2 score measurements, these models had the worst performance in the calibration analysis.

The global interpretability of Shapley values based on a sub-sample was confirmed by the exploratory analysis and the importance of attributes. Age, weighted Charlson score, number of chronic disease diagnoses, number of diagnoses, and number of circulatory system diseases are the most relevant attributes according to those analyses. LIME revealed global interpretability provided fewer relevant attributes and failed to identify weighted Charlson score and number of chronic disease diagnoses as relevant.

The contrast of two extreme cases in the local interpretability analysis evidenced Shapley values provided more detailed explanations, which is in line with previous analyses [35]. The case with a high probability of developing MACE, despite involving an only 19-year-old patient, showed high Shapley values for weighted Charlson score and for several MACE-related diseases (e.g., neoplasms, chronic diseases, metabolic disorders, among others), thus justifying the associated high risk. On the other hand, the low-probability case provided low Shapley values for both weighted Charlson score and number of cardiovascular diagnoses, which explains its low probability, despite involving a 69-year-old patient.

### Strengths

Predictive models were developed in this study with data specific for the Brazilian population of RPMS towards measuring the risk of patients developing MACE. A variety of algorithms was trained and validated, with Random Forest emerging as the one with highest generalization capacity, also confirmed in MIMIC IV dataset.

The interpretability analysis enabled the understanding of the relevance of attributes both globally and individually, which also increase end-user confidence in predictions and identify personalized preventive actions for each patient.

### Limitations and future implications

Although the final model performed very well, more attributes such as medical procedures, lab tests, and medications can be added towards improving it. Additional caution should be taken, since too many attributes can also lead to over-fitting. Another important point to be considered is that plenty of information, such as lab tests, is taken into account for confirming a diagnosis and others, such as medications, may be a consequence of a diagnosis.

Models with greater generalizability can be obtained through multicenter studies that include different hospitals and different regions. However, challenges involve standardization of attributes, authorization to share information, and data quality.

This research is part of the PRECARE-ML consortium and the first stage of model training and validation. The consortium is a multi-center study and will involve model training with the use of federated learning and validation in each partner country.

Although our interpretability analyses showed consistent results both locally and globally, further studies with end-users should be conducted towards the identification and validation of personalized preventive measures for each patient.

In this work we focus on supervised machine learning models for MACE prediction. However, unsupervised methods can also be explored with the advantage of being highly interpretable [36, 37].

Unfortunately, due to the lack of information such as blood pressure, it was not possible to compare our models with traditional risk scores like as the Framingham risk score.

## Conclusions

Among the eight ML algorithms evaluated, Random Forest showed greater generalization power, both internally and externally. Compared to LIME, Shapley values of local interpretability provided more detailed explanations, which is in line with our exploratory analysis and the global interpretability of the model. Machine learning algorithms with proven generalizability and accompanied by interpretability studies should be used to measure individual risks of development of MACE and to provide personalized preventive measures.
